# Overcoming constraints of scaling: Critical and empirical perspectives on agricultural innovation scaling

**DOI:** 10.1371/journal.pone.0251958

**Published:** 2021-05-27

**Authors:** Million Gebreyes, Kindu Mekonnen, Peter Thorne, Melkamu Derseh, Aberra Adie, Annet Mulema, Seid Ahmed Kemal, Lulseged Tamene, Tilahun Amede, Amare Haileslassie, Aster Gebrekirstos, Walter Tamuka Mupangwa, Mohammed Ebrahim, Temesgen Alene, Addisu Asfaw, Workneh Dubale, Simret Yasabu

**Affiliations:** 1 International Livestock Research Institute (ILRI), Addis Ababa, Ethiopia; 2 International Centre for Agricultural Research in the Dry Areas (ICARDA), Rabat, Morocco; 3 Alliance for Bioversity International and CIAT (ABC), Addis Ababa, Ethiopia; 4 International Crop Research Institute for the Semi-Arid Tropics (ICRSAT), Addis Ababa, Ethiopia; 5 International Water Management Institute (IWMI), Addis Ababa, Ethiopia; 6 World Agroforestry Centre (ICRAF), Addis Ababa, Ethiopia; 7 International Maize and Wheat Improvement Centre (CIMMYT), Addis Ababa, Ethiopia; Wageningen University, NETHERLANDS

## Abstract

Scaling is a ubiquitous concept in agricultural research in the global south as donors require their research grantees to prove that their results can be scaled to impact upon the livelihoods of a large number of beneficiaries. Recent studies on scaling have brought critical perspectives to the rather technocratic tendencies in the agricultural innovations scaling literature. Drawing on theoretical debates on spatial strategies and practical experience of agricultural innovation scaling in Ethiopia, this paper adds to the current debate on what constitutes scaling and how to overcome critical scaling constraints. The data for the paper came from a qualitative assessment using focus group discussions, key informant interviews, and document analysis on scaling work done in Ethiopia by a USAID-funded research for development project. The paper concludes with four broad lessons for the current understating of agricultural innovation scaling. First, scaling of agricultural innovations requires a balanced focus on technical requirements and associated social dynamics surrounding scaling targets, actors involved and their social relations. Second, appreciating the social dynamics of scaling emphasizes the fact that scaling is more complex than a linear rolling out of innovations towards diffusion. Third, scaling may not be strictly planned; instead, it might be an extension of the innovation generation process that relies heavily on both new and long-term relationships with key partners, trust, and continuous reflection and learning. Fourth, the overall implication of the above three conclusions is that scaling strategies need to be flexible, stepwise, and reflective. Despite the promises of flourishing scaling frameworks, scaling strategies it would appear from the Africa RISING experience that, if real impact is to be achieved, approaches will be required to be flexible enough to manage the social, processual and emergent nature of the practice of scaling.

## Introduction

Scaling is a major preoccupation of research for development actors in the agricultural sector [1–3]. In the agricultural innovation literature, scaling of innovations is defined as “a deliberate and planned effort to enable the use of innovations to have positive impact for many people across broad geographies” [4]. Scaling of agricultural innovation is seen as an important step in transition from pilots to impacts at wider level and ensuring that investments in the agricultural sector pay off in terms of alleviating poverty and achieving sustainable development goals.

Despite a high-level of interest, however, both the science and practice of scaling are still in early stages of development [5, 6]. Drawing on theoretical debates on spatial strategies in scaling and practical experience of a research for development project in Ethiopia—Africa Research in Sustainable Intensification for the Next Generation Ethiopian Highlands project (Africa RISING)—this paper adds to the current theoretical and empirical debate on what constitutes scaling and how to overcome critical scaling constraints.

From a theoretical point of view, in scaling literature, it is possible to implicitly and explicitly observe a link with multi-level perspective (MLP) approaches for sustainable transition [7–9]. The MLP literature argues that innovations and changes happen at the intersection of the niche, regime, and landscape levels. The niche level provides an incubation platform for change to happen, the landscape level provides impetus and pressure for regime disturbance. Destabilization of the regime creates windows of opportunity for change to happen at the niche level [10]. However, the MLP approach has been criticized for not taking spatial perspectives seriously [11–13]. Spatial perspectives on scale problematize the current treatment of niches, regimes and landscapes to underscore their relational, network and power-laden tendencies [14]. To the knowledge of the authors, the study of Herman et al. [11] is the only study which treats scaling with explicit spatial attention. They have shown that scaling local innovations are embedded in multi-level spatial scales. Within each level, multiple actors are involved in their spread and diffusion. As local innovations move between scales, they adapt and transform. In their travel across scale, they also need to overcome politics and power-related constraints [11].

We build on the ideas of Herman et al. [11] in explaining the multi-level processes of scaling out and scaling up of agricultural innovations. While Herman et al. [11] focus on mature innovations with traceable trajectories of scaling out and scaling up, our theoretical focus enables us to look at innovations that are still in the process of being scaled out and scaled up. In addition, Herman et al. [11] base their theoretical argument on socioecological transformation literature; we use geographical studies of scale and scaling which has enabled us to explain scaling in more social terms.

From an empirical point of view, recent studies on scaling provide complementary critical perspectives to the rather technocratic tendencies in the agricultural innovations scaling literature. Woltering et al. [15] note that current interest in scaling is trapped in the notion of ‘reaching out to many’, while there is an urgent need for a scaling approach that would lead to a ‘new norm’ involving changes in multiple overlapping systems. In the same vein, Low and Thiele [16] show the complexity of scaling in practice by presenting the case of orange-fleshed sweet potato scaling involving a complex interplay of technical, organizational, leadership, and institutional dimensions over a twenty-year period. Totin et al. [17] call for consideration of both material and social practice aspects of agricultural innovations in which scaling needs a good balance of push and pull approaches; the former being technology-orientated and the latter institutions-oriented. A deeper, critical look by Roo et al. [18] at the scaling of innovations in malt barley production in Ethiopia demonstrates that, unless deliberate care is taken, scaling can lead to the exclusion of some vulnerable groups, such as women, youth and poor farmers.

In this paper, we join the debates above and argue for expansion of the concept of scaling from an innovation/material centred approach to one that more effectively captures the complex social relations and practices involved in scaling. Such an understanding of scaling, we argue, is important to overcome critical power and governance related constraints. The paper aims to share scaling practices, missed opportunities and potential areas of action experiences by the Africa RISING project. In doing so, we aim to draw broader lessons on scaling and propose spatial strategies to overcome scaling constraints.

## Conceptual framework

The rather technical definition scaling provided at the beginning of the paper conceals several socio-political issues, such as the actors involved in generation of innovations, the planning process for scaling and the target groups set to benefit from innovations. It renders scaling as a technical problem that can deliberately be planned and executed. Such definitions do not make explicit the role of power relations among actors involved in innovation generation and scaling and socio-political constraints that may limit the possibility of reaching out to wider beneficiaries (scaling out) and institutionalisation of piloted success stories (scaling up). Social science perspectives treat scales as socially constructed and political. This view moves from the notion of scale as a hierarchically bounded space towards a way of looking at it as the result of social interactions that determine our framing of reality and the material consequences of such a framing [19]. This framing has two consequences. First, it allows the productive integration of hierarchical conceptions of scale into a network based formulation that captures the involvement of state and non-state actors in scale making [20]. Second, the social construction of scale invites explicit attention to the role of power relations among actors in an innovation network [12], expanding the focus of scaling from that of innovations *per se* to governance and broad political contexts [2]. The social construction of scale means that actors are able to overcome constraints at a particular level through various rescaling strategies [21, 22]. While it is not within the scope of this paper to deal fully with spatial strategies of scaling, three strategies—scale jumping, scaling down and scale bending—are presented below as they strongly relate to the scaling out and scaling up of agricultural innovations.

Scale jumping refers to the condition in which political power established at one scale is expanded to another [23]. With this strategy, actors expand their influence from local to national, national to regional and regional to global levels. This helps actors to avoid scale traps such as localism, parochialism and particularism through the expansion of their geographical and political reach [23]. An example of scale jumping in the literature is when, at the end of the 1990’s, New York community gardeners came together with state level gardening networks in order to overcome the threat of losing their gardens to real estate expansion. Reframing local gardens as economic engines, environmental buffers and aesthetic resources helped local gardeners gain much needed political support from state level key actors [24]. It is important to note that scale jumping is not a gradual rescaling, but a deliberate attempt to reach out to a higher level in order to achieve aims that would be impossible at lower levels. Hence, scale jumping “reframes” local issues in terms of regional, national or even global interests. For the scaling of agricultural innovations, scale jumping is important when there are critical constraints such as finance, capacity and political legitimacy at the local level, which can only be resolved through resource mobilization or advocacy at higher decision-making levels.

Scale jumping strategies need to be accompanied by scaling down strategies. Scaling down means localizing high-level strategies in order to embed them in cultural and place-based interests [23]. It refers to a form of devolution in which higher level actors engage local actors in order to get sufficient space and support to implement their intended action. Social phenomena are related to place and cultural attachments [25], which suggests that scale jumping needs to complement its broadening strategies with scaling down strategies, in order to be able to implement decisions and influences achieved at a higher levels [23]. An example of a scaling down strategy in the literature, again related to community gardening, comes from Switzerland. It relates to a grassroots movement for community gardening which faced serious critics from its funding agency for not having a strong local orientation in its gardening approach. To address this, the grassroots movement engaged with a neighbourhood youth organization and started educational programs for neighbourhood schools allowing them to use its gardens for educational purposes [26].

Scale bending refers to the spatial strategy of systematically challenging and upsetting the assumptions that relate to particular political activities and a particular scale [24, 27]. It elaborates actions taken by individuals and social groups to challenge and undermine existing arrangements which tie particular decision-making to certain scales [28]. One example of scale bending in the literature comes from Nepal, where local politicians avoided bureaucratic hurdles in their government-led village development groups by working with NGOs for climate adaptation decisions which fell under the jurisdiction of village development groups. For the scaling of agricultural innovations, scale bending means finding alternative mechanisms for surmounting or even resisting market, regulation and policy related constraints faced by local communities. Hence, scale bending strategies could take scaling of agricultural innovations to new areas, activating a different set of scaling strategies, such as advocacy and empowerment, in addition to the conventional ones, such as partnership and capacity building.

In this paper we used the notion of scaling as a social construct and overcoming of scaling constraints as a spatial strategy to unpack the social constituents of scaling, scaling practices and ways of overcoming scaling constraints in Africa RISING project. The paper addresses three inter-related research questions and sub questions (see Table 1). The first research question addresses the social construction of scaling, including the ways innovations are generated, the targets for scaling and the actors involved. The second research question investigates scaling practices, looking closer into the observed processes of scaling and constraints to it. The third research question addresses issues related to overcoming scaling constraints. These research question brings an explicit attention to the social dynamics of scaling and are intended to help develop a better understanding of scaling and scaling processes, tackle scaling constraints and refine scaling strategies.

**Table 1:** Research questions and sub-questions.

What are the social constituents of Africa RISING project scaling work?	What are the scaling practices of Africa RISING project?	What strategies could help to overcome scaling constraints of Africa RISING project?
How were innovations ready for scaling generated? What are the scaling out targets? What are the designed scaling up strategies? Who are the actors involved in the scaling process?	How do scaling out and scaling up processes evolve in practice? What are the scaling out and scaling up constraints observed?	What are the observed practices of scale jumping, scaling down and scale bending strategies? What are the missing opportunities? What are the potential action areas?

## Methodological approach

### Description of the case study

**Fig 1:**
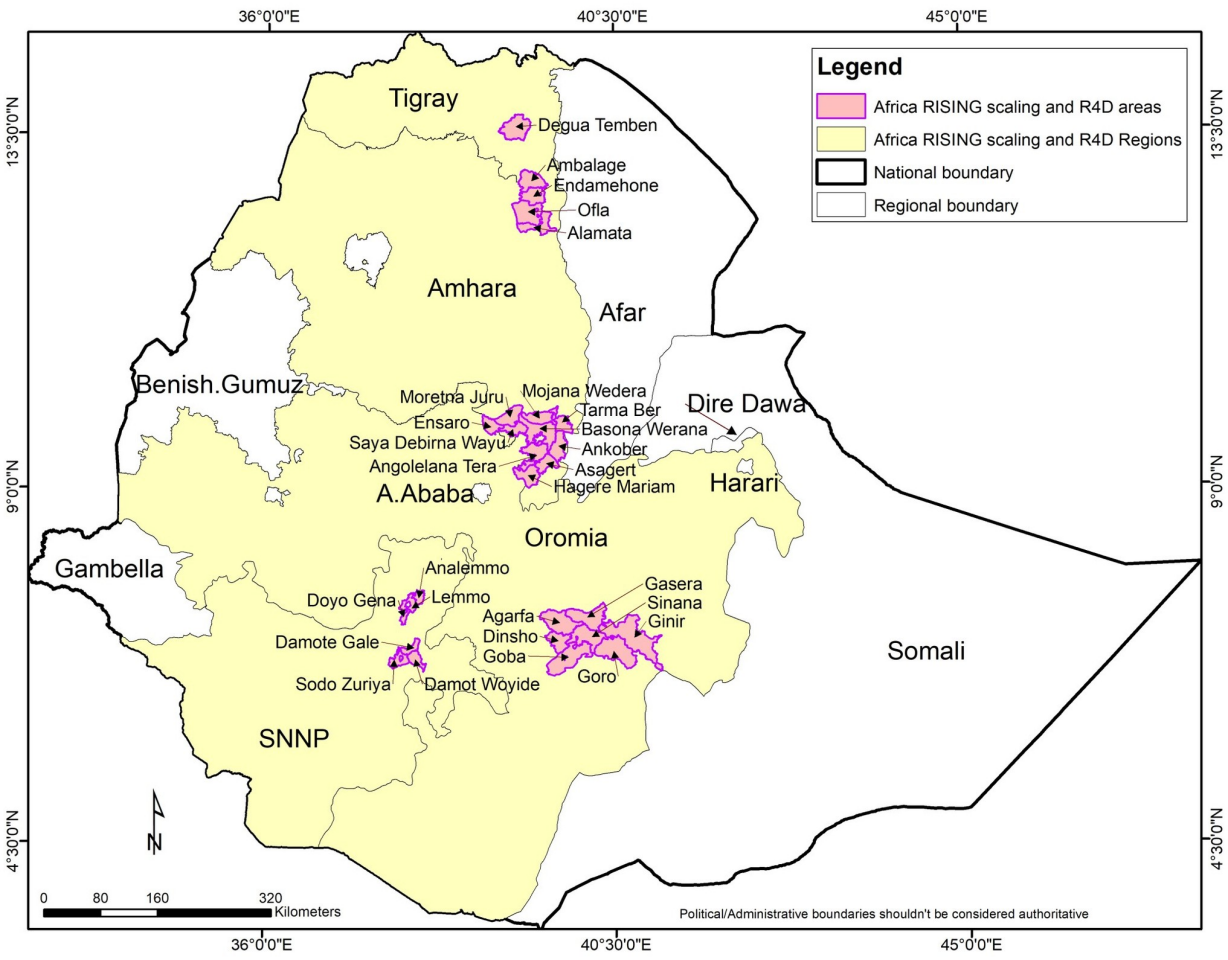
Africa RISING action and scaling intervention sites.

The Africa RISING project focuses on sustainable intensification (SI) of crop-livestock systems in wheat-based farming systems in the Ethiopian highlands. The project has been implemented in two phases since 2011 in four regions of Ethiopia—Amhara; Oromia; Tigray; and Southern Nations, Nationalities and Peoples (SNNP) (Fig 1). The project involves nine Consultative Group for International Agricultural Research (CGIAR) centres, each contributing to different, but linked, parts of the overall research agenda. In Phase I (October 2011–September 2016), the project identified, adapted, validated and deployed farming innovations for SI, generating an evidence base to share with scaling or development partners. The project conducted researcher managed action research and training on farmer fields in four kebeles of four regions. Kebele is the lowest administrative unit in Ethiopia’s government structure.

In Phase II (October 2016–September 2021), the aim has been to facilitate the scaling of innovations validated in Phase I to other areas with similar socioecological conditions through development partnership arrangements. However, there are no explicit guidelines for identifying scalable innovations ex-ante for Phase II. Rather, Africa RISING researchers, together with farmers, local public extension offices and local development partners, have identified scalable innovations using innovation platforms with implicit guiding principles. These include: a) an evidence base for “measurable” SI benefits across the SI domains; b) evidence that predictable trade-offs and unintended consequences and trade-offs have been explored and neutralized; c) clear alignment with development priorities of potential scaling partners; d) resources available to development partners to promote technologies to end-users; and e) evidence that these technologies are attractive to end-users.

### Sampling, data collection and analysis

This paper used both primary and secondary data. The primary data collection involved key informant interviews (KIIs) and focus group discussions (FGDs) (Table 2). At the planning and scientific leadership level, six key informants were interviewed from CGIAR centres based in Addis Ababa, Ethiopia who were involved in the two phases of the project. At grassroots level, four Africa RISING site coordinators were interviewed. Three FGDs were held with zonal and woreda (a woreda is an Amharic word for an Ethiopian administrative organizational equivalent of a district) level scaling partners in Tigray and SNNP regions. The FGD participants were focal persons and scaling partners for Africa RISING, mainly including staff from the offices of agricultural and livestock development. Focal persons are experts who support documentation and tracking of beneficiaries of Africa RISING validated innovations. In SNNP region, two zone-level and four woreda focal persons from the offices of agriculture and livestock development participated in the FGD. In Tigray region, four focal persons from two of the scaling woreda agriculture and livestock development offices participated in the FGD. All except one of the interviews were conducted in Amharic. English was used for one of the KIIs. In order to preserve the privacy of the scaling partners whose cases are presented, the results from the four Africa RISING scaling sites are anonymized in this paper. Guiding questions used for the FGDs were:

- Which innovations, validated by the Africa RISING project, have been scaled?
- To what extent have the innovations been scaled out?
- How did the innovations perform when they were scaled out beyond the original research sites?
- What affects the scalability of different innovations in different places?
- When can we say that scaling is institutionalized at different scales and with different organizations?
- In what ways did actors from regional and federal levels support the scaling process or how could they support it?
- Are there instances where scaling had to go against government rules and regulations? What can be done to improve scaling?

**Table 2:** Primary data collection.

Method of data collection	Number	Participants	Role in scaling
Key informant interview	6	CGIAR (ICRAF, ICARDA, ILRI, IWMI, ICRSAT) researchers based in Addis Ababa	Innovation generation; scaling backstopping research
	4	Africa RISING site coordinators in Tigray, Oromia, SNNP and Amhara regions. The site coordinators are full time employees of the project and are permanently stationed at their respective sites.	Coordinate research for development activities; coordinate activities of local scaling partners
Focus group discussion	3	Hadiya zone office of agriculture and Lemo woreda office of agriculture and livestock development experts in SNNPR; Endamehoni woreda and, Embalaje woreda office of agriculture and livestock development experts in Tigray region.	Coordinate scaling activities of farmers’ organizations; mobilize resources needed for scaling; monitor and follow up field level scaling activities with farmers

The Africa RISING documents and outputs information repository (http://cgspace.cgiar.org/handle/10568/16498) was used as a secondary data source where event reports, blogs and annual reports were collected starting from 2012.

The analysis made use of English transcriptions of the KIIs and FGDs. The data analysis was deductive as the theoretical framework outlined in the first section informed identification of the five codes used to capture the scaling process, namely scaling out, scaling up, scale jumping, scaling down and scale bending. Manual coding was then carried out to categorize the transcription into the five scaling processes. Each of the KII and FGD transcriptions and the secondary sources were read line by line and instances of any of the five scaling concepts, such as facts and figures, testimonials, plans, achievements and challenges were noted. Once story fragments were collected under each of five categories, the analysis continued with the curation of observed practices, missed opportunities and possible areas of actions for each of the five spatial strategies. The final activity in the analysis involved identification of emerging stories under each of five code categories. The analysis went back to the theoretical framework in order to make connections between the empirical scaling experience and the theoretical spatial strategies.

We acknowledge that all authors in this paper are also members of the Africa RISING project team. Whilst all authors tried to genuinely reflect our scaling practice, there is a possibility that this introduced some level of positionality in our findings.

The data and the arguments for the paper were derived from project documents and practical experience of the authors. The human subjects involved in the research were experts at different levels. The data collected from experts was limited to their opinions, with no personal consequences for them. For these reasons, we did not seek ethical clearance for the paper.

## Results

### Scaling in the Africa RISING project

The scaling plan for Africa RISING’s first phase, published in 2015, states that the project would adopt a stepwise approach to scaling with farmers as the starting point for ground-up scaling initiatives [29] The work with individual farmers was expected to expand to cover their kebele and eventually move to other kebeles within the targeted woredas. While the project is mainly focused on R4D, close collaboration with woreda-level agricultural offices was seen as the main mechanism for wider scaling. Within this framework, the project identified seventeen scalable innovations (see Table 3). Long-term and evidence-based relationships with development partners, complemented by trust from a wide range of local actors during the first phase of Africa RISING paved the way for the subsequent deliberate scaling initiative during the second phase [30].

**Table 3:** Examples of Africa RISING scalable innovations, partners and implementation sites.

Research innovations	Thematic areas	Regions	Collaborating CGIAR Centers
Irrigated or rain-fed fodder: oat-vetch mixture, desho grass, sweet lupine, Napier grass, alfalfa, fodder beet	Feed and forage development	Amhara, Oromia, Tigray and SNNP	ILRI, ICARDA, IWMI and CIAT
Fodder and fertilizer trees/shrubs	Improving the efficiency of mixed farming systems through more effective crop-livestock integration	Amhara, Oromia, Tigray and SNNP	ILRI, CIP, ICARDA and ICRAF
Stepwise intensification of faba bean/forage intercropping	Improving the efficiency of mixed farming systems through more effective crop-livestock integration	Amhara and SNNP	ILRI and ICARDA
Crop residue management and utilization: improved storage, choppers and feed troughs	Feed and forage development	Amhara, Oromia, Tigray and SNNP	ILRI and ICARDA
High value fruit trees: avocado and apple	Integration of high value products into mixed farming systems	Amhara, Oromia, Tigray and SNNP	ILRI and ICRAF
Improved management practices and soil test based nutrient amendments: bridging yield gap	Field crop varietal selection and management	Amhara, Oromia, Tigray and SNNP regions	CIAT and ICRISAT
Participatory varietal selection (PVS) on major crops: bread and durum wheat, potato, faba bean, lentil, check pea, food and malt barley	Field crop varietal selection and management	Amhara, Oromia, Tigray and SNNP	CIP, CIMMYT, ICARDA and ICRISAT
Community-based seed production: major crops such as wheat, potato and faba bean	Integration of high value products into mixed farming systems	Amhara, Oromia, Tigray and SNNP	CIP, CIMMYT and ICARDA
Raised bed/ridges and furrow for soil and water conservation (SWC) and agri-intensification	Improved land and water management for sustainability	Amhara, Oromia, Tigray and SNNP	CIP, CIMMYT and ICARDA
Mechanized seeding: seeder fitted on a two-wheel tractor	Field crop varietal selection and management	Amhara, Oromia, Tigray and SNNP	CIP, CIMMYT and ICARDA
Water harvesting, lifting and saving: ponds, rope and washer, solar pumps, and wetting front detector	Improved land and water management for sustainability	Amhara, Oromia and SNNP	ILRI, CIAT, ICRAF and IWMI
Tractors and mounted motor pumps	Improved land and water management for sustainability	Oromia and SNNP	CIP, CIMMYT, ICARDA and IWMI
Management of enset bacterial wilt	Field crop varietal selection and management	SNNP	ILRI, CIP and ICRISAT
Food and seed crop production and storage: groups and cooperatives	Cross-cutting problems and opportunities	Amhara, Oromia, Tigray and SNNP	CIP, CIMMYT, ICARDA and ICRISAT
Integrated model watershed management	Improved land and water management for sustainability	Amhara and SNNP	ILRI, CIAT, ICRAF, ICRISAT, IWMI, ICARDA and CIP

(Source: Adapted from Lunt et al [32]).

Scaling in the second phase of the project aimed at catalysing significant impacts at scale, while retaining the values of Africa RISING as a research project. In its commitment to action research, the project aimed to champion development options that had a solid grounding in high-quality research evidence [31]. The scaling work in the second phase involved working with diverse scaling partners. Thus, the focus of the project shifted from research towards partnership building, including identification of partners, building their capacity and backstopping their work with research to support scaling efforts.

### Scaling out

In the Africa RISING project experience, scaling out has been taken to mean increasing the intensity of use of innovations by individual households, increasing the number of beneficiaries of Africa RISING’s first phase, targeting farmers and kebeles and increasing the target kebeles and woredas with the intention of reaching out large number of farmers with various innovations. The main scaling strategies adopted by the project have been capacity building through training local scaling partners; provision of starter seeds and planting materials, especially for innovations requiring planting materials that are not available locally; and identification of focal persons with each scaling partner who can follow up on the scaling work, documenting and reporting progress. Before each planting season, Africa RISING organizes capacity building and planning workshops in each intervention region. Scaling partners are requested to fill in a form indicating the zones, woredas and kebeles where the partners plan to introduce the innovations, the number of households they expect to reach and what kind of support they expect from Africa RISING (e.g., training, demonstrations and provision of starter planting materials). This information has been used by Africa RISING project site coordinators to plan and execute scaling support strategies. Accordingly, Africa RISING scaling partners reported reaching more than 60,000 farmers in 2016/17 and more than 75,000 in 2017/2018 production seasons [33, 34].

There are some practical insights to be gained from the Africa RISING experience that show the constraints of scaling work. First, scaling out is closely related to the socioeconomic and ecological context of the innovation to be scaled. As indicated by several KIIs, ‘crop innovations generally scaled out much better than livestock innovations’ in all Africa RISING project implementation sites’ (KII with AR site Coordinator). One of the overarching reasons for such a difference is that crop innovations have a better functioning extension system, whereas livestock extension systems are still catching up. “The crop sector has a better extension system, including better organized suppliers of planting materials and experienced farmer organizations, which the livestock sector is still struggling to establish” (KII with Africa RISING site Coordinator). For example, there are well established wheat seed multiplication cooperatives at most of the Africa RISING sites and farmers are willing to pay for wheat seeds. On the other hand, there is less attention paid to forage seeds and those of food legumes. For example, there are no oat and vetch seed production cooperatives, or they have been started only recently. Farmers are also hesitant to spend money on forage seeds.

Secondly, even where innovations are deemed scalable and are ready for scaling out, this does not mean that they will automatically fit everywhere. This is often due to socioecological mismatches between the areas where innovations are generated and the areas they are subsequently targeted for scaling. With respect to improved wheat varieties for example, site coordinators at two Africa RISING sites stated the Africa RISING validated varieties—Mekele 4 and Tsehay—which did not perform as well as the local varieties because the soil type was not the same as in the area where the Africa RISING validated varieties were developed.

A further reason is that in areas where innovations are targeted, local experts and farmers might not be familiar with the innovation and do not have the skills required to maintain its level of performance. The following quotation illustrates this:

“We had a challenge of scaling a forage crop innovation called sweet lupin in our woreda. We were given the seeds to plant without enough knowledge of the crop, both at expert and farmer levels. As a result, many farmers refrained from planting it, and those who planted it did not manage the plant well. We, as experts, were also not in a position to answer technical questions raised by farmers as we had little knowledge on how to plant it properly

(FGD with SNNP region scaling partners).

Thirdly, the rate and extent of scaling out depends on the local availability of planting materials. For example, oat, vetch and tree lucerne were locally available at some of the Africa RISING sites, but not at others. Sweet lupin and fodder beet planting materials were also not available at all sites. “The two cannot be scaled out with the same pace”, said one of the experts who participated in the Tigray region scaling partners FGD. This situation created differences in the performance of scaling out efforts. This insight helps actors participating in the scaling out process to take planning seriously and to make sure that planting materials which have to be brought from other places are made available well in advance. One of the experts who participated in the Tigray region scaling partners’ FGD stated exactly this, “we need serious planning to identify which of the technologies are available and which are not and make sure that we get enough planting materials before the planting season.”

### Scaling up

Scaling up has an institutional dimension. In this case, institutionalization means that Africa RISING validated innovations are included in the annual plans of public extension system actors at various levels and conditions are created for innovations to be widely used. This affects the annual targets of woredas, zones or even regions. When the innovations are included in the work plans of scaling partners, they will be evaluated by councils that must evaluate the performance and achievement of the targets set. The following quotations show such institutionalization by two of the scaling partners at one of the Africa RISING intervention sites:

“Last year (2017/18 production year) was a transition period. So, we had no chance of introducing the innovations in our plans. This year, we introduced most of the innovations in our plans. Faba bean, Hidasie wheat variety and feed trough are all included in our plan. Field pea, Mekele 4 wheat variety and apple were also included.”

(FGD with Tigray region scaling partners).

“At Emblaje (a woreda in southern Tigray) as well, the same is true. Livestock feed innovations such as oat-vetch mixture are all in our plan. Because it is in our plan, we are now able to evaluate our experts accordingly. The plan is also introduced to our woreda council. So everything is institutionalized”

(FGD with Tigray region scaling partners).

Once innovations are scaled up or institutionalized, they allow continuity of the scaling out process. In an FGD with scaling partners at one of the Africa RISING sites, a participant said,

“Now, if Africa RISING phases out, we remain with the innovations. They are now at a stage of no return. Even if we decide to drop some of the innovations, farmers will demand to get them. So we may not have new innovations but the ones we have will remain part of our extension service delivery,”

(FGD with SNNPR scaling partners).

One example of scaling up at the regional level in the SNNP region is the work on avocado varieties. Africa RISING obtained grafted seedlings of five improved avocado varieties—Hass, red 30, Nabal, Ettinger, and Fuerte—from a local horticultural nursery and distributed these for evaluation to a group of Africa RISING farmers. The farmers planted the improved varieties in 2014 with strong support from the project. Subsequently, they purchased further grafted seedlings from Butajira horticultural nursery in 2015. The improved varieties introduced by Africa RISING produce fruits within 1–2 years. They are short, making harvesting very easy, and they are productive. Recently, the SNNP regional government identified two Africa RISING validated avocado varieties—Hass and Ettinger—for the export market and there is high-level regional government support for rural communities to grow these two avocado varieties which can be exported to outside markets and benefit farmers.

However, scaling up, meaning inclusion of Africa RISING innovations in annual plans of partners, has happened only in a limited number of cases, according to the information obtained from site coordinators and scaling partners. There have been various constraints for this. First, partners are hesitant to put scaling figures in their annual plans because it will hold them accountable if they do not achieve the target. Hence, they give scaling plans to the Africa RISING project site coordinators, but these figures are not reflected in their annual plans. “They do not have problems to give us numbers. Each year they give us plans to reach huge number of farmers, but often that is not reflected in their organizational plans,” (KII with Africa RISING site coordinator). Second, scaling partners, particularly those operating within the public extension system, do not have enough incentives for including Africa RISING innovations in their planning. Hence, Africa RISING site coordinators found that the scaling partners they engaged with complained that they are asked to do Africa RISING work without personal incentives. “They often express this both implicitly and explicitly,” said one of the Africa RISING site coordinators (KII with Africa RISING site coordinator). Third, the uncertainty of Africa RISING funding means that even when scaling partners are willing to include Africa RISING innovations in their planning, implementation can be difficult because the available resources are only known at a very late stage, often just before the planting season begins.

### Scale jumping

Scale jumping is an essential strategy for scaling out and scaling up. In the Africa RISING case, the niches where innovations happen, such as woredas, needed scale jumping to higher levels in order to scale effectively and influence regime changes. Some Africa RISING validated innovations, such as crop varieties and some forage species, had strong local extension system and quasi-private actors involvement in their dissemination. Other innovations, for example, livestock related innovations such as the improved feed trough, have no established extension system and no involvement of private sector actors. Scaling of the latter is dependent on strong extension and support system interventions, which are resource intensive. In the public extension system, local resources, especially where there are no special government programs, are stretched to accommodate the additional operational budget required to scale Africa RISING validated innovations. Hence, scale jumping is essential to tackle resource constraints which affect local scaling. When the partners themselves use scale jumping, this involves creative ways of linking up with high-level agendas. The scaling partners often use what we could call ‘narrowing down’ the regional targets to match the woreda interests. For example, FGD participants stated, “if the regional target includes introducing livestock feed, we would fit in oat, vetch, tree lucerne, and other Africa RISING innovations. If the target is introducing better livestock feed management scaling, we would include the feed shed and feed trough”. If it is improved seed, Africa RISING partners would include particular varieties introduced by the project. One scaling partner said:

“By naming Africa RISING innovations, we keep to the regional plan and make it more concrete. That is why we often say Africa RISING innovations do not require additional resources. If planned well, they can all be introduced as part of our work. But this is only our own initiative. The region does not know about these initiatives. Had the region known about it, they would have allocated budget, use it to evaluate service providers and support us in the process. So far, we have not had any problem with the region because there are no problems associated with the innovations. If we have a problem, the region will accuse us of introducing innovations without the approval of the regional government”

Other scale jumping strategies suggested by scaling partners are designed to appeal to higher level policy makers and fit in within their targets. Examples cited included the diffused light store, feed trough and feed shed innovations which require some carpentry skill and could be packaged as rural youth job creation mechanisms. The use of natural resource management (NRM) as a hook to sell some of the Africa RISING innovations was also recommended by one FGD group. For example, the feed trough and feed shed innovations could be effectively linked with the need to avoid free grazing and promote zero grazing. One of the scaling partners said:

“Our woredas are drought prone. As a result, livestock feed is a major problem. In an effort to get enough feed resources for their livestock, farmers tend to use marginal areas to graze their livestock species. The feed trough and the feed innovations of Africa RISING could save us a lot of trouble. They can help preserve our NRM base by reducing feed wastage and promoting efficient use of feed. Hence, if the regional government would take this seriously, it could subsidize the construction of feed sheds and feed troughs for farmers. This would help both the farmers and the environment”

(FGD with Tigray region scaling partners).

When Africa RISING researchers use scale jumping, it is often either to gain high-level political support, or to mobilize resources which are not locally available, or both. Typical examples of Africa RISING scale jumping experiences are related to fertilizer application, solar pumps and small-scale mechanization. A research initiative led by ICRISAT during the first phase showed that fertilizer response is dependent on landscape conditions and the slope of farm plots, which led researchers to recommend differentiated fertilizer rates instead of the widely used blanket recommendation. However, scaling out findings was not immediately possible as fertilizer recommendations are decided at a national level. One of the Africa RISING researchers said, “our fertilizer recommendation is a complex matter to scale. Our work was with farmers on the ground. However, fertilizer-related decisions are made at higher level, where the flow and quantity of fertilizer is decided upon in a top down fashion” (KII with Africa RISING CGIAR researcher). Hence, the researchers took the matter of applying fertilizer rates based on soil maps to a national-level initiative under the auspices of the Agricultural Transformation Agency (ATA) and Ministry of Agriculture (MoA). This policy engagement led to the refinement of ATA’s recommendation, as well as institutionalization (scaling up) of the recommendation by inserting it into the national soil strategy and developing decision-support tools. Another Africa RISING initiative, led by IWMI during the first phase, found that the solar pump innovation for irrigation water lifting was effective in helping farmers to improve their productivity. However, the initial cost of the innovation was beyond what the local partners could afford. Hence, the researchers opted to scale jump to national level by working with actors such as the Agricultural Transformation Agency, the Ministry of Agriculture and International Fund for Agricultural Development (IFAD), who are interested in finding energy-efficient water lifting innovations for small-scale irrigation. The small-scale mechanization work, organized by CIMMYT also had to partner with the Ministry of Agriculture, Mechanization Directorate, in order to get the resources needed to support the scaling out of the two-wheel tractor business model developed during the first phase.

Despite these positive experiences, opportunities for scale jumping have not always been identified and taken advantage of within the overall Africa RISING engagement strategy. Africa RISING engagements have been mainly limited to kebele and woreda levels, and to a small extent, zonal levels. Although regional government experts have encountered Africa RISING products on various occasions, the engagement was not institutional. “It would have been good if we had stronger relations with regional actors, especially the regional extension system,” stated one of the Africa RISING site coordinators (KII with Africa RISING coordinator). Another one added, “having a planning workshop, or even a pre-planning workshop with high-level regional and zonal decision makers is essential,” (KII with Africa RISING site coordinator). Partly, this is because a full-fledged second phase scaling plan was not envisaged during the first phase. Hence, engaging with regional governments was seen as being of lesser importance. In addition, for at least two of the Africa RISING sites, the regional government seat is far from the project areas, which brought logistical challenges to engage with experts on a regular basis. These challenges need to be overcome by intensifying the level of engagement with regional government experts responsible for extension, scaling and inspection.

### Scaling down

Scaling down refers to the process of scaling through embedding resources and policy support gained from scale jumping to local interests. Two examples of scaling down from the Africa RISING experience are related to the fertilizer recommendation and small-scale mechanization discussed in the previous section. Once the location-specific fertilizer recommendation was accepted as best practice at national level and integrated into the national soil strategy, scaling continued with further partnerships that would take the recommendations to local applications. This involved partnering with the GIZ Integrated Soil Fertility Management project and regional and woreda bureaus of agriculture to test the recommendations in more woredas and with more crops.

“Our initial work with Africa RISING created an interest among many national actors. ATA wants to take the recommendations at scale. GIZ helped us test it in different locations,”

(KII with Africa RISING CGIAR researcher).

The small-scale mechanization business model using two-wheel tractors is also expected to go to 16 woredas across the country through the MoA. One important observation from the Africa RISING experience of scaling down is that innovations may travel to geographical areas which are well beyond the initial targets set by the project.

### Scale bending

Scaling partners and Africa RISING site coordinators were asked if there were instances where they had to decide against regional government directions or local politicians in order to scale a particular innovation. One of the areas involving scale bending is the regional seed regulations which demand the use of seeds that are traceably certified, or seeds produced by local seed production cooperatives. Local seed systems are still at a formative stage to implement these regulations, while the demand for seeds is high. At one of the Africa RISING sites, the regulation is functional, whilst at other sites it is on its way. In the region where it is functional, over the last two years, and especially the last year, there have been stringent regulations in respect to the local seed system. Abiding by the regulations brought challenges as both the formal and the semi-formal seed systems were not able to supply seeds demanded by farmers. However, there are alternative channels, within the regulations, that can be employed to ensure the flow of quality seed. For example, in the 2018/19 production season, there was a company which offered to buy malt barley and Africa RISING provided the seed from elsewhere. The result of this initiative was widely appreciated. Many people, including representatives of the regional government, some arriving even without prior notice to scaling partners, came to see the results. One of the scaling partners said:

“Often, there are no problems as long as there are no failures. But if an innovation fails, the regional government would put us in trouble. There are some strict rules and regulations by the regional government. We cannot violate that. But there is some room for scale bending. As stated above, sometimes the regional government experts would not explicitly mention some of the innovations that need to be disseminated. We use our own discretion to introduce new innovations which we feel would benefit farmers. When they see good results they appreciate it a lot and include it in their next year plan,”

(FGD with Tigray region scaling partners).

Another example of scale bending from one of the Africa RISING sites involved Africa RISING site coordinators who had to circumvent unrealistic demands from local politicians. In at least three of the four Africa RISING sites, it was observed that local politicians see Africa RISING as a development NGO. As one of the participants in an FGD put it, “we thought Africa RISING is a big NGO with all the money needed for a large-scale intervention. It is only through time that we learnt that this is a research project with a focus on piloting innovations” (SNNP region scaling partners). Another participant said, “there is a tendency to look at our project as a development project, not a research project. As a result, at times, we face unattainable demand from our scaling partners” (KII with Africa RISING site coordinator). Hence, they expect high-level investment involving many beneficiaries, and support for scaling work with knowledge and provision of materials. Faced with limited budget and a research orientation, the Africa RISING site coordinators avoid direct involvement with local politicians, preferring to work with middle-level experts who appreciate the research orientation and knowledge generation which is the mission of Africa RISING.

## Discussion

Our findings complement the findings of Roo et al. [20], who also argue that scaling agricultural innovations involves both material aspects and social practices. While conventional technology transfer-dominated approaches aim at scaling material aspects of innovations, a focus on social practices emphasizes the complex social relations involved in the scaling process. The social constituents of the Africa RISING project scaling work reveal the importance of material and social aspects of scaling of agricultural innovations. In its first phase of operation (2011–2016), through participatory action research, the project was able to generate scalable innovations which were tested and validated in different agro-ecological settings. The technically sound innovations were well received by local actors because of the trust and cordial relationships between project coordinators and scaling partners. Because of promising commitments from potential partners, the project set out ambitious plan of reaching out to more than half a million beneficiaries in its second phase. The scaling strategy adopted by the project in the second phase has been identification of scaling partners, developing the technical capacity of scaling partners to set scaling targets and integrate Africa RISING validated innovations in their regular work plans and providing backstopping research to help scaling partners achieve their scaling targets. Scaling partners were drawn from a pool of partners who contribute to research prioritisation through participation in diagnostic studies and planning meetings and were able to observe the research process and its results through involvement in innovation platforms (IPs), field days and other engagement mechanisms. In the second phase of the project, the project needed to strengthen the spontaneous scaling which was already happening and shift towards a more deliberate scaling. Hence, the Africa RISING experience is consistent with the multiple prerequisites of scaling, which include but are not limited to, proof of concept and long-term engagement [2].

The scaling strategy Africa RISING worked well. Together with its partners, the project was able to reach out to thousands of farmers with various innovations for sustainable intensification. The scaling practice of the project, however, also reveal much needed insight on constraints of scaling and what might be needed to overcome them. For some of the innovations, absence of well-established extension system and reliable initial planting material suppliers were serious constraints. For other innovations, the high investment cost of innovations and the resource constraints among scaling partners were major limitation to reach out greater number of smallholder farmers. Still for other innovations, ridged policy, regulatory, bureaucratic, and political hurdles constrained possible scaling out and scaling up efforts. These constraints limited the number of smallholder farmers reached by Africa RISING and its scaping partners. That said, the lessons from both the success and failures of the project in tackling these constraints provides important insights for scaling science and practice.

The Africa RISING experience shows that well beyond the technicalities of validated innovations, scalability is often contingent on the scalar politics that define the research project in the first place, and the complex partnerships required for scaling [2]. The fact that the main scaling strategy adopted by the project capitalized on the engagement of local partners in local innovation systems and built their capacity for better achievements meant that the final scaling achievement depended on the scalar politics that the partners found themselves in, such as government bureaucracies, coordination and linkage mechanisms of agricultural extension and development, and multi-level political arrangements.

This paper argues that scalable innovations may not immediately scale out but may need some form of adaptation to the new places that they travel to. This is in line with the findings of Hermans et al. [11], who argue that local innovations may adapt and transform as they travel from their place of origin towards other areas. Hence, strict scaling targets and tracking changes may be difficult to accomplish, and reflection, learning and improving may work better [35]. Scaling up, which denotes institutional changes or alignments that support scaling, is also a complex phenomenon as it involves multiple actors at multiple levels, with differentiated institutional arrangements that affect their functioning. Hence, scaling needs to be mindful of institutional arrangements which set rules, norms and incentives for scaling [36].

In its commitment to elaborate the social dimensions of scaling, this paper identifies strategies to overcome constraints against scaling out and scaling up efforts. When the constraints from scaling out and scaling up arise from local traps, such as resource and capacity limitations, or even resistance from local powerful actors, scale jumping might be necessary. Experience from scaling the Africa RISING interventions shows the importance of scale jumping and of a deeper understanding of and engagement with higher level enabling environments such as regional and national policy, or regulatory mechanisms and programs, and reframing innovations accordingly in order to overcome resource and capacity related constraints [11].

However, scale jumping requires scaling down strategies, as actions at a higher level that are essential for scaling out and scaling up require decision-making at more localized levels. Scaling down often brings resources and capacities from a higher level decision-making space to scale an innovation at local level, which Riddell and Moor also call scaling deep [37]. Experience from Africa RISING shows that while resources mobilized to scale innovations through scale jumping may not necessarily come back to the same places where the innovations were generated, they play an important role in creating an enabling environment for scaling of innovations more widely and in the long run.

An even more political scaling strategy may consider scale bending, a strategy for overcoming limits on scaling out and scaling up efforts set by higher-level decisions. Even in countries where powerful actors such as the state dictate research and development directions, there are always alternative mechanisms to overcome scaling constraints that arise from stringent policies, regulations and market forces. Supporting local scaling partners and farmers to identify such alternative mechanisms without violating policies, rules and regulations requires a politically sensitive mindset to understand safe operating spaces within limiting structural forces at higher levels.

## Conclusion

Our results lead us to four conclusions. First, scaling of agricultural innovations requires a balanced focus on technical requirements and associated social dynamics surrounding scaling targets, actors involved and their social relations. Second, appreciating the social dynamics of scaling emphasizes the fact that scaling is more complex than a linear rolling out innovations towards diffusion. Hence, scaling requires understanding of critical constraints of scaling and the need to addressing them as the scaling process unfolds in practice. The concepts of scale jumping, scaling down and scale bending strategies indicates the importance of addressing power and governance related constraints to scaling. Third, based on our empirical experience, we conclude that scaling may not be strictly planned. Instead, it might be an extension of the innovation generation process that relies heavily on both new and long-term relationships with key partners, trust and continuous reflection and learning. Fourth, the implications of the above three conclusions is that scaling strategies need to be flexible, stepwise and reflective. Despite the promises of flourishing scaling frameworks, scaling strategies it would appear from the Africa RISING experience that, if real impact is to be achieved, approaches will be required to be flexible enough to manage the social, processual and emergent nature of the practice of scaling.

## Supporting information

S1 File Memo of interview with Tigray region site coordinator. (DOCX)

S2 File Memo of interview with SNNPR site coordinator. (DOCX)

S3 File Memo of SNNPR scaling partners interview. (DOCX)

S4 File Memos on interviews. (DOCX)

S5 File Scaling focal persons FGD Tigray. (DOCX)

S6 File Scaling questions. (DOCX)

S7 File Reflections on scaling interviews. (DOCX)

## References

[1] Menter H, Kaaria S, Johnson N, Ashby J. Scaling Up. In: Pachico D, Fujisaka S, editors. Scaling up and out Achieving widespread impact through agricultural research. Cali, Columbia: Centro Internacional de Agricultura Tropical; 2004. pp. 9–24.

[2] Hartmann A. Scaling Up Agricultural Value Chains for Pro-Poor Development. In: Linn J, editor. Scaling up in agriculture, rural development, and nutrition. Washington, DC: International Food Policy Research Institute; 2012.

[3] IFPRI, IITA, ILRI. Africa Research in Sustainable Intensification for the Next Generation Program proposal for a second phase, 2016–2021. International Food Policy Research Institute, International Institute of Tropical Agriculture, International Livestock Research Institute; 2016. https://cgspace.cgiar.org/bitstream/handle/10568/77114/AR_phase2_program_proposal.pdf?sequence=6&isAllowed=y T4—Submitted to: United States Agency for International Development (USAID) by the International Food Policy Research Institute, International

[4] CGIAR. Scaling glossary. Scaling Brief. Bonn: Deutsche Gesellschaft für Internationale Zusammenarbeit; 2020. https://cgspace.cgiar.org/handle/10568/110632 Y3–09.03.2021

[5] Hartmann A, Linn J. Scaling Up. Working Paper. A Framework and Lessons for Development Effectiveness from Literature and Practice Wolfensohn Center for Development, Working Paper. 5. https://www.brookings.edu/research/scaling-up-a-framework-and-lessons-for-development-effectiveness-from-literature-and-practice/

[6] Sartas M, Schut M, Stoian D, Velasco C, Campilan D, Thiele G, et al. Scaling readiness: Accelerating the scaling of RTB interventions. Scaling Readiness Newsletter Series #1. https://cgspace.cgiar.org/handle/10568/89446.

[7] Muilerman S, Wigboldus S, Leeuwis C, Muilerman S. Scaling and institutionalization within agricultural innovation systems: the case of cocoa farmer field schools in Cameroon the case of cocoa farmer field schools in Cameroon. International Journal of Agricultural Sustainability, 2018; 16:2, 167–186

[8] Wigboldus S, Hammond J, Xu J, Yi Z-F, He J, Klerkx L, et al. Scaling green rubber cultivation in Southwest China—An integrative analysis of stakeholder perspectives. Sci Total Environ. 2017;580:1475–82. 10.1016/j.scitotenv.2016.12.126 28038875

[9] Wigboldus S, Klerkx L, Leeuwis C, Schut M, Muilerman S, Jochemsen H. Systemic perspectives on scaling agricultural innovations. A review. Agron Sustain Dev. 2016 8 9;36(3):46.

[10] Geels FW, Schot J. Typology of sociotechnical transition pathways. Res Policy.2007;36(3):399–417.

[11] Hermans F, Roep D, Klerkx L. Scale dynamics of grassroots innovations through parallel pathways of transformative change. Ecol Econ. 2016;130:285–95.

[12] Hansen T, Coenen L. The geography of sustainability transitions. Environ Innov Soc Transitions. 17:92–109.

[13] Coenen L, Benneworth P, Truffer B. Toward a spatial perspective on sustainability transitions. Res Policy. 41(6):968–79.

[14] Raven R, Schot J, Berkhout F. Space and scale in socio-technical transitions. Environ Innov Soc Transitions. 2012;4:63–78.

[15] Woltering L, Fehlenberg K, Gerard B, Ubels J, Cooley L. Scaling—from “reaching many” to sustainable systems change at scale: A critical shift in mindset. Agric Syst. 2019;176:102652.

[16] Low JW, Thiele G. Understanding innovation: The development and scaling of orange-fleshed sweetpotato in major African food systems. Agric Syst. 2020;179102770. 10.1016/j.agsy.2019.102770 32127727 PMC6961970

[17] Totin E, Van Mierlo B, Klerkx L. Scaling practices within agricultural innovation platforms: Between pushing and pulling. Agric Syst. 2020;179102764.

[18] De Roo N, Almekinders C, Leeuwis C, Tefera T. Scaling modern technology or scaling exclusion? The socio-political dynamics of accessing in malt barley innovation in two highland communities in Southern Ethiopia. Agric Syst. 2019;174:52–62.

[19] Marston SA. The social construction of scale. Prog Hum Geogr. 2000;24(2):219–42. Available from: 10.1191/030913200674086272

[20] Jessop B, Brenner N, Jones M. Theorizing sociospatial relations. Environ Plan D Soc Sp. 2008;26(3):389–401.

[21] Reed MG, Bruyneel S. Rescaling environmental governance, rethinking the state: A three-dimensional review. Prog Hum Geogr. 2010;34(5):646–53.

[22] Swyngedouw E. Scaled Geographies. In: Sheppard ES, McMaster RB, editors. Scale and geographic inquiry. Nature, society, and method Oxford: Blackwell; 2004. pp. 129–153.

[23] Jones JP. Scale and anti-scale. In: Richardson D, Castree N, Goodchild MF, Kobayashi AL, Liu W, Marston RA, editors. The international encyclopedia of geography. People, the earth, environment, and technology.Wiley Blackwell, 2017.

[24] Smith N. Uneven development. Oxford: Blackwell; 1984.

[25] Escobar A. Culture sits in places. Polit Geogr. 2001;20(2):139–74.

[26] Ernwein M. Framing urban gardening and agriculture: On space, scale and the public. Geoforum. 2014;56:77–86.

[27] Smith N. Scale Bending and the Fate of the National. In: Sheppard ES, McMaster RB, editors. Scale and geographic inquiry. Nature, society, and method. Oxford: Blackwell Pub.; 2004. pp. 192–212.

[28] MacKinnon D. Reconstructing scale. Prog Hum Geogr. 2010;35(1):21–36.

[29] ILRI. Afica RISING in the Ethiopian Highlands. Addis Ababa: International Livestock Research Institute; 2015. https://cgspace.cgiar.org/bitstream/handle/10568/59799/AR_ethiopia_scaling_2015.pdf?sequence=1&isAllowed=y T4—Scaling Plan Y3–11.23.2018 M4—Citavi

[30] Pound B, Tolera A, Matsaert H. Report of the internally-commissioned external review of the Africa RISING project in the Ethiopian Highlands. Addis Ababa: International Livestock Research Institute; 2015. https://cgspace.cgiar.org/bitstream/handle/10568/66567/AR_Ethiopia_review_may15.pdf?sequence=3&isAllowed=y Y3–11.23.2018

[31] ILRI. About us | International Livestock Research Institute. 2021. https://www.ilri.org/about-us

[32] Lunt T, Ellis-Jones J, Mekonnen K, Schulz S, Thorne P, Schulte-Geldermann E, et al. Participatory community analysis: identifying and addressing challenges to Ethiopian smallholder livelihoods. Dev Pract. 2018;28(2):208–26.

[33] ILRI. Africa Research in Sustainable Intensification for the Next Generation Ethiopian Highlands project Technical report, 01 April–30 September 2018. Addis Ababa: International Livestock Research Institute; 2018. https://cgspace.cgiar.org/bitstream/handle/10568/93237/AR_ethiopia_report_mar2018.pdf?sequence=1&isAllowed=y T4—Submitted to: United States Agency for International Development (USAID) Y3–12.21.2018.

[34] ILRI. Africa Research in Sustainable Intensification for the Next Generation Ethiopian Highlands project Technical report, 1 April -30 October 2017. Addis Ababa: International Livestock Research Institute; 2017. https://cgspace.cgiar.org/bitstream/handle/10568/89909/AR_Ethiopia_report_Oct2017.pdf?sequence=5&isAllowed=y T4—Submitted to: United States Agency for International Development (USAID) Y3–12.21.2018.

[35] Linn J. Overview: Pathways, Drivers, and Spaces. In: Linn J, editor. Scaling up in agriculture, rural development, and nutrition. Washington, DC: International Food Policy Research Institute; 2012.

[36] Kohl R. Addressing Institutional Challenges to Large-Scale Implementation. In: Linn J, editor. Scaling up in agriculture, rural development, and nutrition. Washington, DC: International Food Policy Research Institute; 2012.

[37] Moore A. Rethinking scale as a geographical category. Prog Hum Geogr. 2008;32(2):203–25.

