# Supplementary material for: Overcoming constraints of scaling: Critical and empirical perspectives on agricultural innovation scaling
Source: PLoS One. 2021 May 27;16(5):e0251958. doi: 10.1371/journal.pone.0251958 (PMC8158990; doi:10.1371/journal.pone.0251958)
Supplement: S1 File — (DOCX) [file pone.0251958.s001.docx]

With Mohammed

How was scaling organized, how it different from the first phase

Moha: First phase was technology validation

Second phase, it is more scaling and limited 4rd, in the existing villages

Scaling means, in the research kebeles and beyond

Scaling started n 2017

Technolog9ies selected from the first phase, both on crop, feed management

On wheat, Mekele 4 or Hidasse

HB1307 Barley

Gebericho and Hidosha, Fava Bean

Bilalo Field Pea

Oat Vetch

Feed trough and shade

Desho Grass

Malt Barely,

Tree Lucern

These are scalable technologies, in Africa RISING Tigray areas, are the once who are supposed to improve productivity

The focus is in one Zone, in four districts.

The approach was , planning meeting, training and capacity building for key partners

It was well organized training on all the technologies, with fact sheets showing the end result, the production process and all necessary meterials.

Scaling 2 types, type Idirect seed support for technologies whose sources are not within the region

Type II, linking partners for technologies whose source is within the region. In the second type, partners already proved the significance of some of the technologies in their hands. Hence we stat down for joint plan on some of the technologies for scaling up

Technology type, how much we could reach, what should be the role of Africa rising, and what comes from partners. Based on our provision, partners plan their activities. The initial plan in 2017 was ambitious. The actual implementation was limited. First there was huge financial expectation from AR. There was also input shortage especially from technologies which were found within the region. In technologies that AR brought the inputs from outside, everything went very well.

Scaling was done using clustering approach, one crop for one village

When we brought technologies from outside, our idea was to put it for community seed production. However, our success was limited by two factors. One, there was limited capacity of the inspection unit to come on time and inspect our production. Two, when the seed comes it is supposed to be basic or pre-basic. The one we got tend to be more certified seed. As a result, it could not go into seed production. The seeds do not come with the required documentation with regard to its source. Only the wheat seed passed the seed inspection, hence could not go to seed production. The rest was scaled up using farmer to farmer seed exchange.

Some of the technologies performed very well, even as good as or even better than the validation time.

There were also some technologies scaled up by the partners. Some wheat, oat veth, tree lucern, desho grass, were scaled up by partners. Partners had the seed source here within their reach. They got training on the potentials of the technologies at the start of the project. This was the only support, together with the planning period. They haven’t got any other benefit, but they went into implementing it. One strategy that we used was having a contact person in each district, who would steer the activities of AR. They would make sure that AR technologies are included in the annual plans of their organization. That helped a lot in making sure that AR technologies are well captured. The contact person is different from a focal person. A focal person is often expensive. A contact person does the same thing like focal person, but it is less expensive;

So the strategies for local partners are

Technology introduction

Planning

Contact persons who would steer the process

Working closely with the local leadership

The technologies that we were able to recollect

2 q lentil

3 q wheat

And these were given to farmers as a revolving seed.

The first time we gave it to farmers we gave them the seed and we were expecting them to give us back the amount they took

This time the regional government instructed us that farmers should not get free seeds either in kind or otherwise, as this could create dependency. They rather want farmers to get by purchasing. We sat with a union in the area. We gave the seeds to the local unions for free and the union sold them with lower price.

This year as well, we took lessons from last year. Last year the plan was to reach out 50,000 or so farmers. But the final result was not much more than 8000. This year the plan was around 8,000 and the final result was 6000.

We gave them

Fava bean 10 q

Field pea 10 q

And wheat is already in union and they provided 12q

And oat vetch

Partners also scaled up

Desho

Oat vetch

Feed trough and feed shade, also were scaled up. For example GRAD produced 80 feed trough in 8 villages

Hidasse, we haven’t brought them seed, but they scaled it out widely

HB1394, scaled out largely

Tree lucern, was widely scaled out as well.

**Scaling Out**

Scaling Up

Well, scaling up means institutionalization. However, institutionalization may not be a formal regulation by the district or local partners. If we see institutionalization in its lose sense of regularity and continuity, some of our technologies are at the stage already. For example, feed trough is highly valued and it is being now expanding, even well beyond Africa RISING sites such as in Axum, Adwa. It is now a technology which will not be regressed back.

**Scale jumping**

This year we also worked on malt barley. Our work in a village called Ayba, is well recognized even at regional level. It came at a time when the region was looking for best practice to promote. It will go straight into the seed system. ISSD representatives from Mekele University are well astonished with the result. We also have Raya Beer here which requires high supply of malt barely. The district brought two, three times field days with their own cost, involving the regional government as well. [This sounds like scale jumping rather]

**Scale Bending**

Scale bending is not an easy thing. The government partners participate in all our meetings. It is hardly possible to scale bend from the government regulations and ways of doing things.

There are some technologies that depart from the covnvetial extension. For example, Oat vetch, used to be promoted as a separate crop. We promoted oat vetch planting together. Now they are planting it together.

**Ideal Scaling**

- Starter seed
- Basic and pre-basic seeds provisions
- Capacitating the inspection

A one season intervention needs to work closely with regional inspection team. They would ask whether there is demand or not, we would say we have demand. Then brining basic and pre-basic seeds and planting it through farmers’ seed producers cooperatives would help a lot.

Scaling requires high level involvement of higher level government officials. Some of the technologies could be easily scaled out. If you take feed trough, it could be done with local material if the leadership take it seriously.

There tends to be an extreme sense of dependency on AR. Some of the things could go way far with existing technologies.

The feed trough could help reducing feed lose by 20-30%

The apple disease could be controlled seriously. If the local leadership helps

We need a scaling team

Start with scaling workshop with high level officials, explain them the technologies, and help them to plan for scaling up. Often time, districts may want to include some of the AR technologies but because they are not recognized at regional level, they tend not to be evaluated at it. Hence, having a planning workshop, or even a pre-planning workshop with high level regional and zonal decision makers is essential.
