# Supplementary material for: Overcoming constraints of scaling: Critical and empirical perspectives on agricultural innovation scaling
Source: PLoS One. 2021 May 27;16(5):e0251958. doi: 10.1371/journal.pone.0251958 (PMC8158990; doi:10.1371/journal.pone.0251958)
Supplement: S2 File — (DOCX) [file pone.0251958.s002.docx]

Interview with SNNPR site coordinator, on Scaling

29.11.2018

Technologies scaling well

Avocado went very well

Wheat is also good

On livestock, Oat vetch didn’t go well

These are the top three technologies which are going well

Why these technologies?

For wheat

Scaling requires money. We had NGOs who pay for the scaling, like World Vision

The government seed system is also good, farmers are also willing to pay for the seed of wheat

In the future as well, farmers will pay for wheat, so the government seed system will handle the seed production

For avocado

The project Forest Sector Improvement Project supported our work. Farmers have also been attracted by the high yield of the improved avocado variety.

It could have gone even more, if was not for planting material shortage. The demand is high.

For Oat Vetch

Farmers like lit a lot, they also get some seed. Now farmers are allocating their crop land for fodder production. Oat vetch reach fast and livestock like it a lot.

Tree Lucerne didn’t go as we expect it. There was seed problem which was a result of our delayed delivery of the initial seed

Farmers are not trained to keep the seed, they just feed the whole thing without maintaining seed

The livestock also do not feed it well, it is not very palatable, compared to oat veth

It also dried at many farms

We also need to work a bit more on its utilization, farmers are not well trained on how to feed it to their livestock

Feed trough: Didn’t go fast and autonomously. It needs money to buy the construction material, with the existing model which is expensive, farmers do not pick it up much. Farmers also do not seem to worry too much about the feed wastage. More extension work is needed to convince them that a lot can be saved by using the feed trough.
