# Supplementary material for: Overcoming constraints of scaling: Critical and empirical perspectives on agricultural innovation scaling
Source: PLoS One. 2021 May 27;16(5):e0251958. doi: 10.1371/journal.pone.0251958 (PMC8158990; doi:10.1371/journal.pone.0251958)
Supplement: S3 File — (DOCX) [file pone.0251958.s003.docx]

**SNNP scaling partners’ interview**

**19.11.2018**

Hadia Zone, Livestock Development Office

Hadia Zone, Agriculture Office,

Lemo District, Agricultural Office, Extension Division

Misha District, Livestock Office

Lemo District, Agricultural Office

Lemo District, Livestock Office

Girma: (Lemo District, Agricultural Office): I was in the initial Africa RISING technology verification period

I have seen very good technologies

The Africa RISING technologies support our work

The work in Jawe and Gahna was very good

Farmers participated through out the research process

**For us the experts, it helped us to identify the technology choices of farmers**

There were good works in fruits like avocado, feed and forage

In between there was a bit of a gap

The work did not expand beyond the targeted district

Now the focus is on seed multiplication

This is a good move, as it will reduce the cost of finding seeds from elsewhere

The work on potato was also great

We are not at the centre of scaling providing planting materials for other areas as well

The problem is, we haven’t seen such work expanding beyond the two villages

Fava bean seed shortage has been a serious problem, even the regional government did not have seed

The frequent seed shortage of wheat is also another issues in our area

In the last production serious, we cultivated 6 ha of fava bean in Mish and Lemo districts

Barley also, we cultivate 1 ha of seed

On wheat we plated Hidasse seed, but there was a wheat rust problem which affect most of the plantation. We need more tests to get best performing variety. The wheat intervention also need a full package. There is a chemical support need.

Demeke: We used to bring many seeds and they would fail because of not adapting with the local condition. Farmers used to ask us to bring them verities which works in our areas. Africa RISING answered that question, buy testing verities which work for our context.

Our avocado used to be a local variety, now farmers have access to a better variety

The zone is now working on expanding the work to other farmers

On Food Barely, there was a problem of seed, farmers still use farmers to farmer extension seed produced through AR

Some gaps we observed include: While ICARDA compensates for the PVs work, other CG centres do not do so and that creates problems with farmers

The scaling up is limited to small number of farmers, we need to increase the number of beneficiaries

Farmers also ask for not only feeds and forage, but some more work on improved breeds and animal health

In the new scaling distict, Misha, there has never been a practice of providing fodder seeds, now that starts

There is also capacity building on fodder consumption, with emphasis on use of mix of fodder for better result

Now what is happening are more and more farmers are requesting to have access to the technologies

The seed provision needs to come on time

Otherwise, farmers are changing. They used to think that feed production would compete with crop production, not most farmers dedicate even the whole of their land for feed production

We requested Africa RISING to plant Oat and Vetch, separately, in order to make sure that we retain enough seeds for subsequent production

For Scaling

**On seed that we do not know the source, we need AR to bring us the seed on time**

**On seeds that we have, we need experience sharing, and capacity building on their production an utilization**

**We need more works on extension, more field days, trainings and information sharing mechanisms**

Market is not a problem in our area, when you have the product, either milk or meat, there is enough market. The problem is on the production side

**Scaling out**

**Farmers in the villages where the technologies are generated are well knowledgeable about the technologies, when scaling up with them, it is not a problem. When you have to take the technologies to new places, however, you would need a whole new round of convincing**

Some of the technologies, like sweet lupin, was new, even to experts. Experts do not know much about the technology, they even have some questions and hence was difficult to reach out farmers with such technology

**Overall, there is knowledge gap on the technologies by farmers as well us farmers which needs to be sorted out in the subsequent phase**

Seed is another major problem to scale out: This is true for fava bean, wheat and barely

Scaling out is a resource intensive activity

At first AR is an NGO and it was to bring more resource to reach out to farmers, it is only through time that we understand that Africa RISING is not an NGO and it does things only in small scale

**Scaling is also knowledge intensive, we are now rushing in introducing the technologies. We only give one off orientation or one field day to introduce technologies, while a typical technology may need between 1-3 field days**

If we ask the crop production team about AR technologies, I tell you, only few know about it. Training and capacity building activities are limited to few experts only

Our district have good potential, our farmers are receptive of technologies, and we have well motivated expert staff

The main constraint now is resource,

The district has no resource to properly do the scaling out work

The Lemo District budget was 150, 000,0000 million this year but 143.5 million went into paying for salary. The operational budget of livestock and crop devisions is not more than 20-30,000 birr for the whole year and all activities. The same is true for the Zone. So there is limited possibility of doing meaningful work on AR technologies

Scaling Up

Scaling up requires high-level commitment

The district and zone have no money

There seems to be a lot of resource at regional level, but the region is not responsive to local needs

There needs to be some work to be done by AR in convincing regional and federal level institutions and even donors to invest in AR technologies in Africa RIING intervention areas

Our immediate hope is in Wachamo University. |They have a lot of resource, but because of the leadership reshuffle, they have not been that active

We have no expectation fro the regional government.

A separate discussion with Uni Wachamo Agriculture College Dean: reveals the need to talk with the new administration again, updating them the progress so farm and convincing them to channel some of the community service money that they have to AR related technologies and interventions
