# Supplementary material for: Overcoming constraints of scaling: Critical and empirical perspectives on agricultural innovation scaling
Source: PLoS One. 2021 May 27;16(5):e0251958. doi: 10.1371/journal.pone.0251958 (PMC8158990; doi:10.1371/journal.pone.0251958)
Supplement: S4 File — (DOCX) [file pone.0251958.s004.docx]

Interview with, ICRISAT representative

September 4, 2018, ILRI campus, Addis Ababa

- The IPs were on the ground when I joined in 2015/16. The AR projects were already identified and running well with good coordination of partners. In 2015/16, IPs mobilized all partners and CG centers presented their protocols in IP meetings. Farmers were receptive of our approach partly because the IPs have done a good sensitization work.
- Hence, if I say one important benefit of using IPs for our research, it is the mobilization of farmers to work with us
- The IPs were also essential to disseminate some of our recommendations to other farmers in the area
- The IPs were also essential in reformulating our research questions.
- On scaling: Our fertilizer recommendation is a complex matter to scale up. Our work was with farmers on the ground. However, fertilizer related decisions are made at higher level, where the flow and quantity of fertilizer is decided on top down fashion. Hence, even though the local IPs appreciate our finds, it was hard for them to manipulate their fertilizer recommendations.
- From our protocol perspective future IPs need to have farmers unions, ATA soil section, research and others who are involved in fertilizer value chain.
- I haven’t attended any IP meeting myself, although I was the one responsible for the research on ground. I know Dr. Tilahun attended one or so meeting and Yidnekachew attended another one.
- The Africa RISING approach was innovative. The fact that a number of CG centers went to same villages with their protocols made it possible to approach farmers needs from a holistic point of view. I work on fertilizer, but if someone requests assistance with seeds, there are other CG centres to respond to that. AR didn’t have much money, but the approach was innovative.
- Our initial work with Africa RISING created an interest among many national actors. ATA wants to take the recommendations at scale. GIZ helped us to test it in different locations. These are done not through the IP but through our networks.

Interview with ICRAF representative, ILRI Campus

September 5, 2018,ILRI Campus , Addis Ababa

- ICRAF was working with Africa RISING on high value fruit trees, particularly mango and apple
- Public investment on fruit trees seedling production is limited.
- We provided planting materials
- In other places we go deeper and develop the seedling system, like in Sinana
- In Tigray there is a serious apple disease problem on apple varieties introduced before us. We have done quite a lot in developing viable solutions, but not much was achieved.
- On innovation platforms: I joined ICRAF after the IP were established and had their first meeting. I started attending their meeting starting from the second IP meeting on wards
- I attended most of the IP meetings, I mean the once at district level. The village level IP meetings were mostly organized by the site coordinators with little help from the Africa RISING IP facilitation team
- The IP were useful in tapping up local knowledge and resource. For example in Sinana, the IP members used their own membership initiative to protect the planted seedlings. They would rotate together across their farmers to check the status of the seedlings and penalize those who did not take care of them.
- It also created a sense of competition among them. Imagine you go and work with a single farmer. They could damage whatever you are working on and go with it. Here, the IPs act as a social self-control mechanism to make farmers responsible to the agreement they get into at the initial state of the research process.
- We had 50 beneficiaries from each site for apple and 164 from each site on avodado
- All the IP were very usfeful
- The woreda IPs had institutional strengthen to make decisions that would influence the work we do at village level.
- At village level the once in Sinana were very strong, the same is true with Lemo. The one in Debrebirihan was weak, followed by Maychew.
- The representation of actors in the IP was good enough for us, we had all the relevant actors
- There were some inconsistencies in the meetings, some would not come, some would send different people for the different meetings
- Dr. Aster also participated in few meetings
- The IPs built the capacity of members. It has shown them that the approach is fruitful. However their technical capacity only is not enough to scale up things. They lack the necessary fund to work on some of the recommendations that we left them with. Once we leave the project sites, we also do not return back to check their status.
- There are some autonomous scaling up activities. For example in Sinana, we tried avocado. There was resistance both from within us and others stating that it will not survive in the highlands. But we decided to do a simple trial, not even with proper methodological approach. It did well over time. The district then requested for more seedlings and we provided them with some.

Interview with ILRI representative

August 13, 2018, ILRI Campus, Addis Ababa

- It is not only in Africa RISING, but there is strong interest by ILRI on IPs
- ILRI is determined to reaching out users with evidence for decision making
- We used IPs in other projects are well to reach out to people working on the ground
- IPs are one of the mechanisms that we use to engage with stakeholders
- The IP establishment started with a meeting which brought regional and district experts. [note that the national level is jumped]. The purpose of the meeting was to introduce Africa RISING for our partners.
- We then agreed to establish strategic, operational and research groups.
- To my understanding the main purpose of establishing the IPs was to support Africa RISING project activities. However there was no intention of limiting them to Africa RISING related work only. We were actually encouraging them to use them for other purposes as well.
- The structure of the IPs was taken from the previous experiances of ILRI in different projects
- Since we were interested in working at village and district levels, there was limited interest from our side on establishing regional level IPs
- We were aware that there were other platforms to bring actors together. But for management reasons, we chose to establish our own. However our activities were not in contradiction with that of the others.
- Overall the IPs functioned well. We had good partners both at district and village levels, there were dedicated team of facilitators, and the research projects were activity participating.
- There was an incentive mechanism for participant through capacity building activities and experience sharing
- But there were some constraints as well;
- People have different expectations beyond what the project could afford. Hence some use the IPs to squeeze the project resources in their own interest
- There were also some problems of managing meetings
- The site coordinators were also burdened with lots of responsibilities, coordinating the various protocols
- Overall the private sector actors involvement was poor
- The commitment of the government partners was good
- In SNNP, there was strong NGO involvement
- In Tigray the research centres were very active
- In Debre Birihan, the government institutions were active
- In Oromia, the University was very active
- The facilitation of IPs from the head office side was very good. At site level the site coordinators may lack some facilitation skills. There were some efforts to build the capacity of site coordinators, but still there were some limitations
- The IPs enabled turning evidence into action
- There was an assumption that Africa RISING partners would take up some of the research problems and reesrach agenda
- For example inset disease was raised as an important problem to be addressed by Africa RISING and a protocol was design on it afterwards
- We also took chickpea to Tigray region and IP members reject is it is a crop not known in the area
- Some of the technologies may not survive if AR leaves. But some of it may do, for example the feed trough. Partners are already asking for it.
- Tree Lucern is very well accepted in Debre Birhan, and it could survive without us
- In Bale we introduced potato intercropping and farmers love it already
- Where the IPs failed;
  - There was high expectation by some members of the IP
  - Sustainability is a big issue. Now that the project is over, the IPs are no more functional. They were not holding meetings since we stopped funding. There was an assumption that the government structure would use them after we leave, but that does not happen

Interview with ILRI representative

August 15, 2018, ILRI Campus Addis Ababa

- There was an experience in ILRI already in use of IPs with humid tropics
- Then there was a conusltatnt for Africa RISING IP establishment who did the first study
- The main entry point for us as researchers were the FRGs
- The research agenda were set based on the number of assessments made at filed level
- Then protocols were designed afterwards
- The farmers research group were interest based. The FRGs were then used for IP formation at Kebele levdl
- I still believe that the FRGs were essential for our research work. We were doing research on farmers field and we need some way of mobilizing them, which the FRG did a good job
- Forming the FRG was not expensiv at all. It acutally reduces cost
- But the IP at Kebele and District levels, they have expense
- We had all the actors we need, but their commitment was limited
- District and zonal level actors were very enthusiastic and cooperative during the initial phases of the project
- They were our enabling agents
- We had good reception
- We went through high level officials during our launching, so all was good
- Eventually, however, the IPs role started to be limited to the IP meeting time.
- There was also staff turn-over, limiting the effectiveness of the IPs
- The researcher also ended up focusing on their specific research work
- The initial focus of our work was on testing some technologies. Eventually we realized that we have some good results.
- Our government partners were strong. Our NGO partners and mainly private sector actor partners were very week
- So the FRGs were useful in the day to day running of our research work
- The kebele level IPs were rather used to disseminate some of the lessons learned by the FRG
- The Kebele IPs also challenge some of our research activities and helped us to improvse it. For example the feed trough was initially desined to be moblle. When presented at the IP, farmers said it is better if we make it fixed. They also recomeded addition of storage and shade
- At addis level, there were good facilitators, there was a science forum where CGs regularly meet
- The sustainability of the IP: Is for sure will not continue when the funding ends, as just happened
