# Supplementary material for: Overcoming constraints of scaling: Critical and empirical perspectives on agricultural innovation scaling
Source: PLoS One. 2021 May 27;16(5):e0251958. doi: 10.1371/journal.pone.0251958 (PMC8158990; doi:10.1371/journal.pone.0251958)
Supplement: S5 File — (DOCX) [file pone.0251958.s005.docx]

**Scaling Focal Persons FGD memo**

10/11/2018, Mychew

- [Three participants, Anonymized]

Hadish: Embalaje was not in the first round of AR. We joined during the scaling stage. But it all participatory process. We were presented with the technologies and we chose what suites us. We introduced the technologies into our planning. It was all something which adds value to our plan. it started with what we do and endeavoured to add value to it.

At Embalaje we took crop varieties such as Hidase, which is not included in our annual plan. On livestock we started small and expand with time. Especially on Oat Vetch, we used to plant them separately, now we learned to plant it at the same time. This is now made part of the normal extension strategy. On tree lucern, we got new variety which suited the “dega” area. It is now fully integrated in our seedling production. The feed shade is also a new introduction. We show it during field visit for our farmers and now we introduced it at our FTCs for demonstration. Through GRAD, last year we constructed 10 of it. Once we test the technologies with small farmers, we introduce it to our annual plan.

Hadush: We have been working with Africa RISING since the begning. We took a number of technologies that AR promoted as part of our extension strategy. Most of the technologies are now being dessimated in our Kebeles. Our FTCs are demonstrating most of the technologies.

Nigus: We have many NGOs in our area but the work of AR is different in that it has been doing things that work well with farmers. We have serious animal feed problem and AR is working on that. We know Oat and Vetch in theory, but getting the mix was always a difficulty. The Oat and Vetch mix is not like a discovery for us. Farmers testify to the value of such practice. They observed huge increase in milk production because of this technology. Sweet Lupine is also new to us and introduced by AR. Fodder beat is also the same.

Combined with the feed trough, we are able to provide an alternative feeding system to eradicate free grazing and promote zero grazing. Now farmers are modifying the feed trough using local resources.

In areas where there is access to irrigation, farmers are planting alpha alpha introduced by AR
The tree lucern is allowing poor woman farmers to fatten sheep and support their families. It is also serving us a very good bee forage.

Scaling out:

It is a step by step process. We introduce technologies and scale it slowly. The process needs to be demand driven. The trouble is tracking scaling extent. Often, the things we introduced in small get disseminated through farmer to farmer extension.

**The FTCs play a significant role in demonstrating the technologies.**

Embalaje is new to the scaling activity. As a result there are mixed results. On Wheat, we took it first. However farmers told us that it matures a bit late. Hence, we found it suitable mainly in areas where they can supplement it with irrigation. We learned this lesson after our first attempt.

The feed trough has no problem, so it continued expanding.

Sweet Lupine, farmers did not accept it yet. We planted it at our FTCs and last time with the support from the district we organized field day for 500 farmers. But they asked us numerous questions which we were not able to answer since the technology was not used by farmers yet. We were rather recommended to introduce it to some willing farmers and test the results.

**[This would mean that in the districts that the project is introducing new technologies, it may help a lot to set a little budget to demonstrate the technology, rather than assume that farmers will take it anyways.]**

Some technologies require continuous demonstration, training and testimonies.

The source materials of some of the technologies is easily accessible locally. For example, Oat Vetch and Tree Lucern are available here. Sweet Lupine or Fooder Beat is not available. The two cannot be scaled out with the same pace. Hence we need serious planning, to identify which of the technologies are available and which are not and make sure that we get enough planting material before the planting season. **[This shows the importance of the planning phase well in advance].**

The local extension system is not good at scanning available technologies outside of its immediate reaches. Hence the local research system need a continuous work in introducing different varieties and demonstrate their results. THIS COULD BE A REGULAR WORK OF RESEARCH ORGANIZATIONA AND UNIVERSITIES.

One of the good scaling strategy might be establishing a good link between source materials and local extension systems. An experience sharing visit to technology sources might help speeding up and concretizing planning process.

**Scaling UP**

Something is said to be institutionalized when we are able include it in our annual plans. When something is included in our plan we are able to allocate budget to it and we evaluate our performance accordingly.

Last year was a transition period. So we had no chance of introducing the technologies in our plans. This year we introduced most of the technologies in our plans. Fava Bean treatment, Hidasse, Feed Trough are all included in our plans. Field Peas, Mekele 4 and Apple are also include

At Emalaje as well, the same is true. Animal feed, Oat Veth are all in our plan. Because it is in our plan, we are now evaluating our experts accordingly. The plan is also introduced to our district council. So everything is institutionalized.

Now if AR phases out, we remain with the technologies. They are now at a state of none return. Even if we decide to drop some of the technologies, farmers will demand to get them. So we may not have new technologies, but the ones we have will remain part of our extension delivery.

**Scale Jumping**

Our districts are drought prone. As a result animal feed is a major problem. In an effort to get enough grazing for their livestock, farmers tend to take marginal areas under grazing. The feed trough and the feed technologies of AR could save us a lot of trouble. They can help to preserve our NRM base by reducing feed wastage and promoting efficient use of feed. Hence, if the regional government would take this seriously, it could subsidize construction of feed shades and feed troughs for farmers. This helps both farmers and the environment. **[This is an interesting example of scale jumping. It also shows that actions like advocacy might be a right strategy for scaling]**

The regional government needs to be aware of the technologies. Our plans are often a result of top down target given to us from the regional governments. For now the way we are introducing AR into our plan is through naming some of the generic provisions by the regional government. For example, if it says introducing livestock feed, we would say Oat Vetch, Tree Lucern or some thing. When it says introducing better feed management, we would include the feed shed and feed trough. When they say improved seed, we would say Hidase and others. By naming AR technologies, we keep the regional plan and make it more concrete. That is why often times we said AR technologies do not require us additional resource. If planned well, they can all be introduced as part of our work. But this is only our own initiative. The region does not know about these things. If they knew about it, they could allocate budget to it, use it to evaluate us and support us. So far we do not have problem from the region because there are no problems associated with the technologies. If we are to face a challenge, the region will accuse us of introducing technologies without approval of the regional government.

We can also sell AR technologies at higher levels using rural youth job creation agenda. This is a major issue and we could develop business plans to secure funding.

**One strategy to make sure that the region supports the work might be to call the different directors in the regional bureau of agriculture during the planning meeting that is organized by Africa RISING. AR also need a regional contact person to follow up with the agenda.**

**[Note that scaling was not a major issue during the initial stage of AR. Hence, regional government experts were not involved. That makes it a bit hard not to buy in their support. If they were involved, they could have taken the technologies easily.]**

**Scale Bending**

There are some strict rules and regulations by the regional government. We can not violate that. But there are some rooms for scale bending. As stated above, sometimes the regional government experts would not explicitly mention of some of the technologies that needs to be disseminated. We rather use our own discretion to introduce new technologies which we feel would benefit farmers. When they see good results they appreciate it a lot and take it in their next year plan.

Other times the regional government may have some loop halls when it comes to some regulations. Last time, they were still on the formative stage of formalizing the use of certified seeds that are not introduced through farmers seed producers for Barely. But there was an opportunity to produce through AR support. There was also one company who was ready to take up the produce. We were not able to wait the approval of the regional government and we proceed with our plan. The result was appreciated a lot. Many people, even those from the regional government, some even without our notice came to visit the results and now that is being taken as a bench mark. This are important scale bending activities. Often, there are no problems as long as there are no problems. But if something happens because of our technologies, the regional government would put us in trouble.
