# Supplementary material for: Overcoming constraints of scaling: Critical and empirical perspectives on agricultural innovation scaling
Source: PLoS One. 2021 May 27;16(5):e0251958. doi: 10.1371/journal.pone.0251958 (PMC8158990; doi:10.1371/journal.pone.0251958)
Supplement: S6 File — (DOCX) [file pone.0251958.s006.docx]

**Question Guides for Scaling Assessment?**

1. Who are the actors involved in scaling and why are they interested in joining hands with Africa RISING?
2. Which of the technologies have been under scaling?
3. To what extent do technologies scaled out?
4. How do technologies perform when they are scaled out outside of the original research sites?
5. What affects scalability of different technologies in different places?
6. When do we say that scaling is institutionalized at different scales and with different organizations?
7. In what ways are actors from the regional and federal level supporting/ could support the scaling process?
   - To what extent is their support critical?
   - To what extent are they involved?
8. Are there instances where scaling had to go against government rules and regulations?
9. What can be done to improve scaling?
   1. To increase the number of people who are using technologies?
   2. To institutionalize the technologies in the existing extension system?
   3. To get support of regional and higher level decision making bodies?
   4. To challenge the existing system and scale beneficial technologies?
