# Supplementary material for: Overcoming constraints of scaling: Critical and empirical perspectives on agricultural innovation scaling
Source: PLoS One. 2021 May 27;16(5):e0251958. doi: 10.1371/journal.pone.0251958 (PMC8158990; doi:10.1371/journal.pone.0251958)
Supplement: S7 File — (DOCX) [file pone.0251958.s007.docx]

**Some of the reflections on Scaling at SNNPR**

- For us the experts, it helped us to identify the technology choices of farmers

**Scaling Out**

- Material Aspects: The need for a separate strategy for scaling of technologies with planting materials within the area and those with planting material that should come from outside;
  - On seed that we do not know the source, we need AR to bring us the seed on time
  - On seeds that we have, we need experience sharing, and capacity building on their production an utilization
- Knowledge and dissemination aspects:
  - In villages outside of AR sites, the technologies are still new:
  - For scaling partners, with high staff turnover, knowledge on the technologies is limited
  - Hence, there is an overall knowledge gap both by farmers as well as local experts
  - This challenges our two assumptions
    - That we made good introduction of the technologies for our partners
    - That the partners will have the means to train their staff and their clients
- Scaling out is a resource intensive activity: Local resource form the extension system seems completely drained to accommodate more from AR

**Scaling Up**

- The local University has good potential, but suffers from the recent change of leadership
  - This requires revitalization of the relationship with the university as all in the leadership position are new
- Scaling up requires high-level commitment
  - This may require engaging the regional government and some of the NGOs and government flagship programs

Areas for consideration

- More investment on trainings, field days and communication materials is needed to create need and convince farmers to adopt the technologies
- Building local seed system is still the way to go;
  - There is good opportunity as farmers and the local administration are committed to help out
  - This requires starting the process early to identify tailored seed system development strategy for each technology
    - For example, there seems to be good willingness to pay by farmers for crop seeds such as wheat, barely, and fava bean.
- More work needs to be done with to engage the regional government and government flagship programs and Wachamo university to invest in the AR technologies
